# Using Serum Specimens for Real-Time PCR-Based Diagnosis of Human Granulocytic Anaplasmosis, Canada

**DOI:** 10.3201/eid2901.220988

**Published:** 2023-01

**Authors:** Carl Boodman, Courtney Loomer, Antonia Dibernardo, Todd Hatchette, Jason J. LeBlanc, Brooks Waitt, L. Robbin Lindsay

**Affiliations:** University of Manitoba, Winnipeg, Manitoba, Canada (C. Boodman);; National Microbiology Laboratory, Winnipeg (C. Loomer, A. Dibernardo, B. Waitt, L.R. Lindsay);; Dalhousie University and Nova Scotia Health, Halifax, Nova Scotia, Canada (T. Hatchette, J.J. LeBlanc)

**Keywords:** anaplasmosis, bacteria, vector-borne infections, zoonoses, *Anaplasma phagocytophilum*, emerging infections, ticks, diagnostics, PCR, Canada

## Abstract

Whole blood is the optimal specimen for anaplasmosis diagnosis but might not be available in all cases. We PCR tested serum samples collected in Canada for *Anaplasma* serology and found 84.8%–95.8% sensitivity and 2.8 average cycle threshold elevation. Serum can be acceptable for detecting *Anaplasma* spp. when whole blood is unavailable.

Human granulocytic anaplasmosis (HGA) is a tickborne infection caused by the intracellular bacterium *Anaplasma phagocytophilum* (*1*), an emerging pathogen in North America (*2*–*5*). HGA can manifest as a subclinical infection; however, most symptomatic persons have fever, myalgia, and headache associated with thrombocytopenia, leukopenia, and elevated transaminase levels (*3*,*6*). Although uncommon, multiorgan failure and death occur predominantly in elderly and immunocompromised patients or when treatment is delayed (*7*,*8*). The manifestation of HGA as a nonspecific febrile illness can lead to lack of recognition, and delays in antimicrobial administration can cause illness and death (*9*,*10*). Early diagnosis is essential to avoid these preventable complications.

Laboratory diagnosis of HGA can be established by using microscopy, serology, or nucleic acid amplification test (NAAT) (*3*,*11*). Microscopy can be used to diagnose acute infections, but relies on experienced personnel to visualize intragranulocytic clusters or morulae in peripheral blood (*3*,*11*). Because morulae are present in only 25%–75% of cases, microscopy lacks sensitivity (*6*,*9*). Serology is more commonly used to diagnose HGA, relying primarily on indirect immunofluorescence assays (IFAs) (*7*,*9*). However, serologic tests are often negative during the first week of symptoms and require paired acute and convalescent serum samples >2 weeks apart to improve sensitivity (*8**–**10*). NAAT can be performed to detect *A. phagocytophilum* in whole blood or buffy coat and is the preferred test during the first 2 weeks of illness (*9*,*10*). However, most persons evaluated for tickborne infections have serum samples submitted as their primary specimen because serology is the standard diagnostic method for Lyme disease, the most common tickborne infection in North America. Unless anaplasmosis is considered when the patient is first seen, a whole blood specimen is rarely available. We used residual serum samples submitted for *Anaplasma* sp. serology in Canada to determine if serum samples could be an acceptable alternative to whole blood for the diagnosis of HGA by real-time PCR.

## The Study

We tested 2 different serum specimen groups for *A. phagocytophilum* DNA. The first group consisted of serum samples from persons who were positive for *A. phagocytophilum* by using the NAAT of whole blood. The second group consisted of acute and convalescent serum samples (drawn >2 weeks apart) submitted to the National Microbiology Laboratory (Winnipeg, Manitoba) for *Anaplasma* serology during 2020–2021. The samples were anonymized, and the investigators were blinded to serology results. Ethics approval was not required because anonymized samples were evaluated for a quality improvement study.

We isolated DNA from 100 μL of serum by using DNeasy 96 kits (QIAGEN, https://www.qiagen.com) and eluted the DNA in 100 μL of elution buffer. We used carrier RNA (Applied Biosystems/Thermo Fisher Scientific, https://www.thermofisher.com) to improve recovery of low amounts of nucleic acids. We used T4 bacteriophage DNA as a positive extraction control. We amplified the *msp2* gene of *A*. *phagocytophilum* as previously described (*12*) by using 5 µL of template DNA in 30 µL reaction volumes containing TaqMan Universal Master Mix (Applied Biosystems). We performed amplifications on a ViiA7 system (Applied Biosystems) and thermocycling conditions were as follows: 2 min at 50°C, 10 min at 95°C, and 40 cycles of 95°C for 15 s and 60°C for 1 min. We included synthetic *A*. *phagocytophilum* DNA (Integrated DNA Technologies, https://www.idtdna.com) as a positive control and master mix without DNA as a negative control in each run. A sample was considered positive if cycle threshold (Ct) values were <40. We reextracted and retested positive samples to ensure reproducibility. Samples with repeated Ct values of <40 were considered positive. Positive samples with insufficient volume for reextraction were considered positive. We calculated averages and ranges from the initial extraction.

We used the semiquantitative Focus Diagnostics *A*. *phagocytophilum* IFA IgG kit (DiaSorin, https://www.diasorin.com), and IgG titers >1:64 indicated current or previous *A*. *phagocytophilum* infection (*13*). We defined seroconversion as a >4-fold increase in titer between acute and convalescent serum samples.

Of the 33 specimens from the first group of serum samples (Table 1), we collected 23 serum samples on the same day as whole blood and 10 serum samples on a different day. The maximum time between serum and whole blood sampling was 8 days. We collected whole blood samples before serum samples for 2 patients. PCR showed 28 (84.8%) serum samples were positive for *A*. *phagocytophilum* of which 6 (18.1%) had an IFA titer >1:64. The average Ct values were 27.6 (range 17.7–39.5) for whole blood and 30.4 (range 19.9–38.8) for serum samples. Among 5 patients who had PCR-positive whole blood samples but PCR-negative serum samples, the average Ct was 35.1. All 10 serum specimens collected on a different day were PCR positive. We tested an additional 90 paired whole blood and serum samples, and the tests showed 95.8% sensitivity (Appendix Table 1).

**Table 1:** Comparison of PCR values for serum and whole blood samples in study using serum specimens for real-time PCR-based diagnosis of human granulocytic anaplasmosis, Canada*

Sample no.	IFA titer	Serum Ct values					Whole blood Ct values				∆Ct	Td‡
		Ct-I	Ct-R	Average†	Result		Ct-I	Ct-R	Average†	Result		
1	<1:64	19.9	NS	19.9	Positive		17.7	16.1	16.9	Positive	3	5
2	<1:64	21.8	NS	21.8	Positive		22	21.1	21.55	Positive	0.25	3
3	<1:64	22.8	21.8	22.3	Positive		22.1	21.5	21.8	Positive	0.5	0
4	<1:64	22.9	NS	22.9	Positive		18.5	17.9	18.2	Positive	4.7	0
5	<1:64	23	22.5	22.75	Positive		20.5	19.7	20.1	Positive	2.65	1
6	<1:64	23.3	22.1	22.7	Positive		19.1	18.1	18.6	Positive	4.1	1
7	<1:64	24.2	24.2	24.2	Positive		21.2	20.9	21.05	Positive	3.15	1
8	1:2048	25	25.8	25.4	Positive		22	21.9	21.95	Positive	3.45	0
9	<1:64	26.6	26	26.3	Positive		24.6	25.4	25	Positive	1.3	0
10	<1:64	26.6	25.6	26.1	Positive		23.2	23.5	23.35	Positive	2.75	0
11	1:512	27	NS	27	Positive		24.8	25.3	25.05	Positive	1.95	2
12	1:64	28	25.3	26.65	Positive		28.6	29.2	28.9	Positive	−2.25	0
13	<1:64	28.6	27.5	28.05	Positive		23	22.6	22.8	Positive	5.25	0
14	<1:64	28.8	28.1	28.45	Positive		33	33.3	33.15	Positive	−4.7	3
15	<1:64	29.2	28.3	28.75	Positive		27.7	27.1	27.4	Positive	1.35	0
16	1:2048	29.9	27.4	28.65	Positive		26.9	27	26.95	Positive	1.7	0
17	1:64	30	29.2	29.6	Positive		22	21.8	21.9	Positive	7.7	0
18	<1:64	31.7	31.7	31.7	Positive		30.5	30.2	30.35	Positive	1.35	0
19	<1:64	32.4	33.5	32.95	Positive		26.4	26.2	26.3	Positive	6.65	0
20	1:512	32.8	31.7	32.25	Positive		37	36.5	36.75	Positive	−4.5	8
21	<1:64	36.4	37.8	37.1	Positive		37.8	37.8	37.8	Positive	−0.7	0
22	<1:64	36.5	35.8	36.15	Positive		28.4	28.5	28.45	Positive	7.7	0
23	<1:64	36.7	36	36.35	Positive		32.5	32.7	32.6	Positive	3.75	0
24	<1:64	37.7	36.3	37	Positive		34	34	34	Positive	3	0
25	1:1024	37.8	37.4	37.6	Positive		32	32	32	Positive	5.6	0
26	1:256	38.2	38.3	38.25	Positive		25.1	24.8	24.95	Positive	13.3	−84
27	<1:64	38.3	40	39.15	Negative		37.2	37.6	37.4	Positive	1.75	0
28	<1:64	38.4	35.3	36.85	Positive		24.1	24.2	24.15	Positive	12.7	−4
29	<1:64	38.8	38.2	38.5	Positive		31.3	31.2	31.25	Positive	7.25	0
30	<1:64	40	38.3	39.15	Negative		38.9	39.5	39.2	Positive	−0.05	0
31	<1:64	40	40	40	Negative		35.3	36.7	36	Positive	4	0
32	1:64	40	40	40	Negative		33.4	32.9	33.15	Positive	6.85	0
33	<1:64	40	40	40	Negative		30.9	31.0	30.95	Positive	9.05	0

*Only Ct values <40 after repeat extraction were deemed positive. Ct, cycle threshold; Ct-I, Ct values for initial extraction; Ct-R, confirmatory Ct values for repeat extraction; ∆Ct, difference between average serum Ct and average whole blood Ct; IFA, indirect immunofluorescence assay; NS, no sample remaining for repeat extraction; Td, time difference in days between serum sampling (earlier) and whole blood sampling (later). †Average of Ct-I and Ct-R for each isolate. ‡Negative numbers indicate that whole blood was sampled before serum samples.

Of 154 paired acute and convalescent serum samples submitted for *Anaplasma* serology, 19 (12.3%) acute specimens and 3 (1.9%) convalescent specimens were PCR positive (Table 2). Average Ct values were 30.3 (range 23.7–37.5) for acute samples and 34.3 (range 27.6–39.9) for convalescent samples. We did not observe seroconversion in 10 (52.6%) patients who had PCR-positive acute serum specimens.

**Table 2:** Comparison between PCR-positive acute serum samples and paired convalescent serum samples in study using serum specimens for real-time PCR-based diagnosis of human granulocytic anaplasmosis, Canada*

Sample no.	Acute serum samples					Convalescent serum samples				Time, d†	Conversion‡
	IFA titer	Ct-I	Ct-R	PCR status		IFA	Ct-I	Ct-R	PCR status		
1	<1:64	23.7	24.7	Positive		1:128	40.0	NA	Negative	24	Yes
2	<1:64	24.3	NS	Positive		1:512	35.5	NS	Positive	33	Yes
3	<1:64	25.1	NS	Positive		<1:64	27.6	NS	Positive	42	No
4	<1:64	25.8	NS	Positive		1:1024	39.9	37.1	Positive	13	Yes
5	<1:64	26.8	NS	Positive		1:512	40.0	NA	Negative	56	Yes
6	1:64	28.0	25.3	Positive		1:256	40.0	NA	Negative	49	Yes
7	1:2048	28.2	30.4	Positive		1:2048	40.0	NA	Negative	12	No
8	<1:64	28.8	28.1	Positive		1:128	40.0	NA	Negative	21	Yes
9	<1:64	29.7	NS	Positive		<1:64	40.0	NA	Negative	39	No
10	1:1024	31.0	29.9	Positive		1:1024	40.0	NA	Negative	28	No
11	<1:64	31.2	31.4	Positive		<1:64	40.0	NA	Negative	0	No
12	<1:64	31.7	31.7	Positive		1:256	40.0	NA	Negative	51	Yes
13	1:1024	32.4	32.8	Positive		1:256	39.8	40.0	Negative	8	No
14	<1:64	32.4	33.4	Positive		1:256	40.0	NA	Negative	38	Yes
15	1:512	32.8	31.7	Positive		1:512	40.0	NA	Negative	27	No
16	<1:64	34.7	36.3	Positive		<1:64	40.0	NA	Negative	38	No
17	1:1024	35.6	36.3	Positive		1:512	40.0	NA	Negative	57	No
18	<1:64	36.1	37.8	Positive		1:64	40.0	NA	Negative	10	No
19	<1:64	37.5	36.8	Positive		1:256	40.0	NA	Negative	46	Yes

*Only Ct values <40 were deemed positive. Ct, cycle threshold; Ct-I, initial Ct values (in duplicate); Ct-R, confirmatory Ct values for repeat extraction; IFA, indirect immunofluorescence assay; NA, not applicable; NS, no sample remaining for repeat extraction. †Time difference between acute and convalescent serum sampling. ‡Seroconversion was defined as >4-fold increase in IFA titer between acute and convalescent samples.

Of the 154 paired acute and convalescent serum samples, 28 (18.2%) were serologically positive, but only 11 (7.1%) demonstrated seroconversion (Appendix Table 2). Titers increased from <1:64 to 1:64 in 3 paired samples, 13 samples demonstrated stable or decreasing titers, and 1 titer doubled. PCR of acute samples detected 9 of 11 (81.8%) patients who displayed seroconversion. PCR was negative using acute serum samples for 2 patients; those patient samples had initial IFA titers >1:1024, indicating either previous infection or delayed sampling. The sensitivity of serum-based PCR was 81.8%, and specificity was 93.0% compared with seroconversion (Appendix Table 3).

## Conclusions

Because *A*. *phagocytophilum* occupies an intracellular niche, the prevailing dogma maintains that whole blood or buffy coat specimens are necessary for detection of *A*. *phagocytophilum* by PCR (*9*,*10*). Because serum is commonly obtained when tickborne infection is suspected, serum is a convenient PCR specimen to diagnosis HGA. Compared with whole blood, serum-based PCR has a sensitivity of 84.8%–95.8% and an average Ct elevation of 2.8.

PCR is superior to serology for diagnosing acute HGA (*10*). Few PCR-positive acute serum samples were associated with elevated IFA titers. PCR using acute serum samples resulted in a superior positivity rate (12.3%) than acute seroconversion measurements (7.1%). Acute serum specimens were 6.3 times more likely to be PCR positive than convalescent specimens, indicating the importance of early specimen collection when pursuing molecular diagnosis of HGA (*10*). The sensitivity of serum-based PCR was 81.8%. Although 81.8% sensitivity is comparable to the whole blood dataset, 10 patients with PCR-positive acute samples did not demonstrate acute seroconversion. Antimicrobial administration might have aborted or delayed seroconversion, which has been hypothesized in a previous study (*14*), although no clinical data exist to confirm this hypothesis. Similarly, 2 patients who had negative PCR results for acute serum samples ultimately had seroconversion. We did not have companion whole blood to determine whether those false negatives were the result of decreased sensitivity of serum compared with whole blood or the acute serum was collected after the acute bacteremia stage. Many acute samples had titers greater than 1:512, which suggests those 2 samples were collected after acute bacteremia. Although whole blood remains the optimal specimen for PCR, this study demonstrates that reflex PCR testing of acute serum samples submitted for *A*. *phagocytophilum* serology might improve diagnostic sensitivity for acute HGA when whole blood is unavailable.

Appendix Additional information for using serum specimens for real-time PCR-based diagnosis of human granulocytic anaplasmosis, Canada.
