# Supplementary material for: Using Serum Specimens for Real-Time PCR-Based Diagnosis of Human Granulocytic Anaplasmosis, Canada
Source: Emerg Infect Dis. 2023 Jan;29(1):175–8. doi: 10.3201/eid2901.220988 (PMC9796190; doi:10.3201/eid2901.220988)
Supplement: Appendix — Additional information for using serum specimens for real-time PCR-based diagnosis of human granulocytic anaplasmosis, Canada. [file 22-0988-Techapp-s1.pdf]

Article DOI: https://doi.org/10.3201/eid2901.220988

Using Serum Specimens for Real-Time PCR-Based Diagnosis of Human Granulocytic Anaplasmosis, Canada

Appendix

Appendix Table 1. Contingency table comparing PCR results from 90 additional paired whole blood and serum samples in study using serum specimens for real-time PCR-based diagnosis of human granulocytic anaplasmosis, Canada*

Serum	PCR-positive whole blood	PCR-negative whole blood	Total/row
PCR-positive serum	69	0	69
PCR-negative serum	3	18	21
Total/column	72	18	90

*Whole blood was used as the gold standard. Sensitivity was 95.8% (95% CI 88.3%–99.1%), specificity was 100.0% (95% CI 81.5%–100%), positive predictive value was 100.0%, and negative predictive value was 85.7% (95% CI 66.5%–94.8%).

Appendix Table 2. Comparison between serologically-positive acute serum samples and paired convalescent serum samples categorized according to seroconversion in study using serum specimens for real-time PCR-based diagnosis of human granulocytic anaplasmosis, Canada*

Sample	Acute serum samples				Convalescent serum samples					
no.	IFA titer	Ct-I	Ct-R	PCR result	IFA titer	Ct-I	Ct-R	PCR Result	Td	Seroconversion†
1	<1:64	25.8	NS	Positive	1:1024	39.9	37.1	Positive	8	Yes
2	<1:64	23.7	24.7	Positive	1:128	40	NA	Negative	12	Yes
3	<1:64	28.8	28.1	Positive	1:128	40	NA	Negative	56	Yes
4	<1:64	40	NA	Negative	1:128	40	NA	Negative	50	Yes
5	<1:64	31.7	31.7	Positive	1:256	40	NA	Negative	57	Yes
6	<1:64	32.4	33.4	Positive	1:256	40	NA	Negative	51	Yes
7	<1:64	37.5	36.8	Positive	1:256	40	NA	Negative	30	Yes
8	<1:64	24.3	NS	Positive	1:512	35.5	NS	Positive	10	Yes
9	<1:64	26.8	NS	Positive	1:512	40	NA	Negative	26	Yes
10	<1:64	36.1	37.8	Positive	1:64	40	NA	Negative	38	No
11	<1:64	38.3	40	Negative	1:64	40	NA	Negative	48	No
12	<1:64	40	NA	Negative	1:64	40	NA	Negative	52	No
13	1:64	40	NA	Negative	1:128	40	NA	Negative	50	No
14	1:64	28.0	25.3	Positive	1:256	40	NA	Negative	21	Yes
15	1:64	37.4	40	Negative	1:256	40	NA	Negative	33	Yes
16	1:64	40	NA	Negative	1:64	40	NA	Negative	31	No
17	1:64	40	NA	Negative	1:64	40	NA	Negative	48	No
18	1:64	40	NA	Negative	1:64	40	NA	Negative	49	No
19	1:128	40	NA	Negative	1:128	40	NA	Negative	179	No
20	1:256	40	NA	Negative	1:256	40	NA	Negative	78	No
21	1:512	32.8	31.7	Positive	1:512	40	NA	Negative	20	No
22	1:512	38.4	40	Negative	1:512	40	NA	Negative	46	No
23	1:1024	31.0	29.9	Positive	1:1024	40	NA	Negative	49	No
24	1:1024	32.4	32.8	Positive	1:256	39.8	40	Negative	13	No
25	1:1024	35.6	36.3	Positive	1:512	40	NA	Negative	37	No
26	1:1024	39.8	40	Negative	1:512	40	NA	Negative	39	No
27	1:2048	28.2	30.4	Positive	1:2048	40	NA	Negative	24	No
28	1:2048	40		Negative	1:2048	40	NA	Negative	43	No

*Only Ct values <40 after repeat extraction were deemed positive. Ct, cycle threshold; Ct-I, initial PCR Ct values; Ct-R, confirmatory Ct values after repeat extraction; IFA, indirect immunofluorescence assay; NA, not applicable; NS, no sample remaining to perform repeat extraction; Td, time difference in days between serum sampling (earlier) and whole blood (later).
†Seroconversion was defined as a ≥4-fold increase in titer between acute and convalescent samples.

Appendix Table 3. Confusion matrix using 4-fold increase in antibody titer between acute and convalescent serum samples as the standard in study using serum specimens for real-time PCR-based diagnosis of human granulocytic anaplasmosis, Canada*

Serum	Positive, >4-fold seroconversion	Negative, <4-fold seroconversion	Total/row
PCR-positive acute serum	9	10	19
PCR-negative acute serum	2	133	135
Total/column	11	143	154

*Seroconversion was defined as a ≥4-fold increase in titer between acute and convalescent samples. Sensitivity was 81.8%, specificity was 93.0%, positive predictive value was 47.4%, and negative predictive value was 98.5%.
